# Expansion of Single Chains Released from a Spherical Cavity

**DOI:** 10.3390/polym15010198

**Published:** 2022-12-30

**Authors:** Chia-Cheng Chu, Pai-Yi Hsiao

**Affiliations:** 1 Institute of Nuclear Engineering and Science, National Tsing Hua University, Hsinchu 300044, Taiwan; 2 Department of Engineering and System Science, National Tsing Hua University, Hsinchu 300044, Taiwan

**Keywords:** polymer expansion, scaling theory, molecular dynamics simulations

## Abstract

A two-stage model is developed to explain the phenomena of chain expansion, released from a confining cavity. In the first stage, the chain is assumed to expand as a sphere, while in the second stage it expands like a coil. The kinetic equations for the variation of chain size are derived in the two stages by balancing the rate of the free energy change with the rate of the energy dissipation. Langevin dynamics simulations are then performed to examine the theory. We find that the expansion process is dominated by the second stage and the evolution of chain size follows, mainly, the predicted curve for coil expansion, which depends on the chain length and is not sensitive to the confining volume fraction. It permits to define the expansion time for the process. Further study reveals that the chain does undergo a spherical expansion in the first stage with the characteristic time much shorter than the one for the second stage. As a consequence, the first-stage variation of chain size can be regarded as an add-on to the principal curve of expansion designated by the second stage. The scaling behaviors and the associated scaling exponents are analyzed in details. The simulation results well support the theory.

## 1. Introduction

Packing and unpacking biomolecules are very important processes in cells and microbiology [1,2]. Understanding the mechanisms and dynamics of these processes help researchers in the development of novel nanotechnologies [3,4]. For example, genetic materials, such as DNA or RNA molecules, can be packaged into small particles for applications [5,6]. The particles are delivered to target cells by some ways. After reaching the cells, the packaged substances can be then released for therapeutic repair. This kind of platform mimics the function of a virus, formulating a promising delivery vector for gene therapy [7,8,9].

In nature, a viral genome is protected by a protein shell, called capsid [10,11]. The capsid possesses a predominated structure of icosahedron, such as in herpesvirus [12] and adenovirus [13], or helix, such as in the influenza virus [14] and coronavirus [15,16]. It can be further enveloped or wrapped in a lipid membrane to form a nanoparticle. The entry of virus to a cell is effectuated by membrane fusion or endocytosis [10,11]. Uncoating of the capsid is triggered later in the cell interior by low endosomal pH and promoted by proteasome activity [17,18]. The DNA or RNA chains are then released to proceed replication. Recent studies revealed that icosahedral viruses expel few pieces of pentamers on the capsid to enable genome release [19,20]. Further study showed that the release pathways can be classified into two main categories: in a rapid release pathway, the capsid ruptures and opens a big hole to allow quick release of the genome in a microsecond order; while in a slow release pathway, the genome escapes through a crack on an edge or at a vertex of the capsid and milliseconds are needed to complete the process [21]. In addition to lowering the pH, capsid uncoating and genome release can be also triggered by increasing temperature [22,23].

Theoretical investigation of the release process mainly focuses on the ejection of a DNA/RNA chain from an enclosed cavity through a narrow tube [24,25,26,27,28,29,30]. It aims to explain the infection mechanism of another type of virus, called phage [10,11]. A phage consists of a polyhedral capsid head and a helical tail, and has its genome stored in the head. It infects a host cell by injecting the genetic chain through the tail tube to the cell interior. No deformation of the capsid head is required in the process. Recently, our group developed a theory which divides an ejection process into two main stages: a confined stage and a non-confined stage [31,32]. For a confined-stage dominated process, the ejection time $\tau_{\mathrm{ej}}∼N^{\frac{2}{3\nu }+\frac{1}{3}}\phi_{0}^{-\frac{2}{3\nu }}$ is predicted while for a non-confined-stage dominated one, $\tau_{\mathrm{ej}}$ scales as $N^{1+2\nu }$. The theory unifies the polymer ejection with the problem of polymer translocation [30,33,34], and is able to explain the two phenomena under one theoretical framework. In addition, non-negligible time is spent for the heading monomer to traverse the tube, in order for a real start of the ejection [31,32]. This new stage is independent of the two main stages and can be regarded as a nucleation process. The whole theory has been generalized from a three dimensional space to a two-dimensional one, and validated [35].

Theoretical study concerning the dynamics of a genetic chain release via capsid uncoating is relatively few in literature [36,37]. To tackle the problem for the first step, we can simply consider the process as a free release of a chain from an encapsulation. This kind of study may look basic. However, it provides the necessary foundations for a further development of more complicated and realistic theories, such as restricted release of chain from a big open hole, hindered release impeded by uncoated pieces of capsid pentamers, and so on, in the future. Pitard and Orland [38] have investigated the swelling/collapsing behavior of a Gaussian chain being quenched into a good/bad solvent condition. The swelling kinetics were predicted: $R^{2}\left(t\right)≃R_{0}^{2}\left(1+{\left(\frac{t}{\tau_{c}}\right)}^{\frac{3}{4}}\right)$ for time *t* smaller than the Rouse time and $R\left(t\right)≃R_{\mathrm{eq}}\left(1-\exp\left(-\frac{t}{\tau_{1}}\right)\right)$ in the larger *t* regime, where $R_{0}$ and $R_{\mathrm{eq}}$ are the initial and the equilibrated chain size in the bulk solution, respectively, and $\tau_{c}$ and $\tau_{1}$ are the characteristic time. Yoshinaga [39] studied the unfolding kinetics of a collapsed semi-flexible chain and suggested a three-step process: swelling, disentanglement, and relaxation. The evolution of chain size was predicted to scale as ${\left(1+\frac{t}{\tau_{i}}\right)}^{\alpha_{i}}$ with $\tau_{i}$ being the characteristic time, and the kinetic exponent $\alpha_{i}$ was found to be $\frac{1}{5}$, $\frac{1}{8}$, and $\frac{1}{4}$, respectively, in the three steps. Later, in collaboration with Sakaue [28], they extended the work by assuming that the chain keeps a spherical shape in the expansion and balanced the free energy change with the energy dissipation via Stokes frictions around the blobs; the size variation for a flexible chain was thus given by $R\left(t\right)≃R_{0}{\left(1+\frac{t}{\tau_{0}}\right)}^{\alpha }$ with $\alpha =\frac{3\nu -1}{9\nu }$. Using a similar approach of uniform spherical expansion, Mitra and Kundagrami [40] studied swelling of single polyelectrolyte. Accounting for charge regularization around chains, they predicted fast swelling kinetics for polyelectrolyte at high temperature, low dielectric mismatch, and low salt concentrations. Lee et al. [41,42] have investigated the swelling of a long-collapsed polymer released in a good solvent condition and found that the dynamics depends on the degree of self-entanglement inside of the globule. For an entanglement-free wet globule, the predicted chain size swells similarly to $R\left(t\right)∼t^{\frac{1}{5}}$ and the characteristic time is about $N^{\frac{3}{2}}$. For a strongly-entangled globule, the chain stays long time in an arrested state, exhibiting some specific correlations, and the time to escape the arrested state is about $N^{2}$. Tang et al. [43] have applied uniform electric fields to collapse DNA molecules and studied their expansion by switching-off the fields. They suggested two expansion pathways: for unentangled DNA, the chains expand continuously as $R_{\mathrm{eq}}^{2}-R^{2}\left(t\right)∼\exp\left(-\frac{t}{\tau }\right)$, while for self-entangled DNA, the chains expand in distinguishable steps and the chain grows similarly to $R_{a}-R\left(t\right)∼\exp\left(-\frac{t}{\tau_{a}}\right)$ in an arrested state. Collapse of single polymers and its counterpart action, expansion, have also been investigated by changing the solvent quality via quenching and sudden rising of temperature in experiments [44,45,46,47]. Researchers have found that expansion of an aged globule takes much longer time than a non-aged globule, owing to the formation of tight knots in the aged one [46,47]. A stretched exponential growth $R^{2}\left(t\right)=R_{\mathrm{eq}}^{2}-\left(R_{\mathrm{eq}}^{2}-R_{0}^{2}\right)\exp\left[-{\left(\frac{t}{\tau }\right)}^{\beta }\right]$ has been used to analyze the experimental data [45,46]. Because knots have great influences on the behaviors of chain [48,49,50,51], many efforts have been devoted to the study of the formation of knots [52,53] and the dynamics and interaction of knots on a chain in free solutions [54,55,56], in flows [57,58], and in confined spaces [59,60,61,62,63,64].

Comparing to the ejection, an expansion phenomenon shall be much faster and stochastic because a collapsed chain swells in all directions, not just along the particular (tube) direction like in ejection. It renders the expansion process less predictable. Only through a large number of sampling of the data, expansion behaviors can be investigated in a reliable way by ensemble analysis. Moreover, experiments of single chain swelling are quite difficult and elaborated, requiring many efforts and techniques for controlling and observation. Computer simulations, on the other hand, provide an alternative approach to investigate chain expansion in a simple and repeatable way under well-controlled conditions. In this work, we aim to understand the kinetic details of single chain expansion released from a confining cavity. A two-stage theory is developed in Section 2, which assumes a spherical expansion first, followed by a coil-like expansion in the second stage. We then perform extensive Langevin dynamics simulations to verify our theory by varying the chain length and the confining volume fraction. The model, setting, and procedure of simulation are described in Section 3. For each studied case, 1000 independent runs are performed to ensure sufficient acquisition of data for accurate analysis. The results are presented in Section 4. We first study the mean evolution of chain size during an expansion process in Section 4.1. The scaling behaviors of chain size in the second stage (the dominant stage) is then analyzed in Section 4.2. Section 4.3 studies the change of chain conformation from a sphere-like state to a coil state. The first-stage expansion is then analyzed in Section 4.4. The speed of expansion is calculated in Section 4.5 and compared with the theory. Finally, we give our conclusions in Section 5. The variations of chain size are scaled by using the two characteristic times for the two stages. A picture of chain expansion is proposed and the scaling behaviors are discussed.

## 2. Theory of Polymer Expansion Released from a Confining Cavity

We study the kinetics of a polymer freely released from a confining spherical cavity. The chain is modeled by a bead-spring chain with both the bead diameter and the bond length equal to σ. We assume that the number of monomers on the chain is *N* and the cavity diameter is *D*. The volume fraction of the chain in the confining cavity is, thus, $\phi_{0}=N\sigma^{3}/D^{3}$. To study free expansion, the confining wall is suddenly removed at the starting point and no external impedance intervenes to affect the dynamics of chain relaxation. This model can serve as a primary model to understand genome release of DNA or RNA chain from a ruptured virus capsid [19,20,21,65].

When a releasing process begins, the compressed chain expands very quickly. The expanding conformation of chain is assumed to be spherical as proposed by Sakaue and Yoshinaga [28]. With advance of the time, the chain has to transform its conformation to a coil in order to attain its final structure in a bulk solution. We, therefore, develop a two-stage theory to describe the process. In the first stage, the relaxation undergoes with a spherical expansion. The kinetic equation can be derived by balancing the rate of the free energy change of the system with the rate of the energy dissipation occurred during the process, and reads as

(1) $$\frac{dF}{dt}=-\eta N{\left(\frac{dR}{dt}\right)}^{2}$$

where *F* is the free energy, η is the friction coefficient, and *R* is the size of the chain. We have assumed the Rouse dynamics because the fluid can flow through the chain. The free energy for such a spherical polymer of size *R* can be estimated from the blob theory [66,67,68] and reads as

(2) $$F∼k_{B}TN^{\frac{3\nu_{b}}{3\nu_{b}-1}}{\left(\frac{R}{\sigma }\right)}^{-\frac{3}{3\nu_{b}-1}}$$

where $\nu_{b}$ is an exponent, describing the scaling relation $\xi_{b}∼\sigma g_{b}^{\nu_{b}}$ in a blob which has size $\xi_{b}$ and contains $g_{b}$ monomers. Plugging the free energy expression into Equation (1), we are able to solve the differential equation, and the evolution of chain size is predicted:

(3) $$R\left(t\right)=R_{0}{\left(1+\frac{t}{\tau_{s}}\right)}^{\alpha },$$

where $R_{0}$ is the initial chain size, $\alpha =\frac{3\nu_{b}-1}{6\nu_{b}+1}$ is the exponent, and $\tau_{s}=a_{s}\left(\frac{\eta \sigma^{2}}{k_{B}T}\right){\left(\frac{R_{0}}{\sigma }\right)}^{\frac{1}{\alpha }}N^{\frac{-1}{3\nu_{b}-1}}$ is the characteristic time with $a_{s}$ being a prefactor. Because the chain is initially confined in a small cavity, the chain segments in blobs should be in a melt state in the beginning. Therefore, $\nu_{b}≃0.5$ is expected [67].

In the second stage, the chain continues to expand but the conformation turns to be coil-like. The Flory free energy should be used now and reads as [66,67]

(4) $$F∼k_{B}T\left[\frac{R^{2}}{N\sigma^{2}}+\frac{v_{\mathrm{ex}}N^{2}}{R^{3}}\right]$$

where $v_{\mathrm{ex}}$ is the excluded volume of a monomer. Using the free energy, the kinetic equation is deduced from Equation (1). It is a non-linear, Bernoulli-type, ordinary differential equation [69]. The variation of chain size can be solved exactly and the result is

(5) $$R\left(t\right)=R_{F}{\left(1-b_{c}\exp\left(-\frac{t}{\tau_{c}}\right)\right)}^{\frac{1}{5}}.$$

Here, $R_{F}$ is the size of a relaxed chain in the bulk solution and $\tau_{c}=a_{c}\left(\frac{\eta \sigma^{2}}{k_{B}T}\right)N^{2}$ describes the characteristic time for the chain expansion in the second stage with $a_{c}$ being a prefactor. The parameter $b_{c}$ should be 1 according to the scaling analysis shown later from our simulations.

## 3. Model and Setup

We perform molecular simulations to verify our theory. The chain is modeled by a bead-spring chain: each bead represents a monomer and there are *N* beads on the chain. The excluded volume interaction of the monomers is described by the Weeks–Chandler–Anderson (WCA) potential [70],

(6) $$U_{\mathrm{ex}}\left(r\right)=\left\{\begin{array}{cc} 4\varepsilon \left[{\left(\frac{\sigma }{r}\right)}^{12}-{\left(\frac{\sigma }{r}\right)}^{6}+\frac{1}{4}\right] & \mathrm{for}\;r\leq \sqrt[{6}]{2}\sigma \\ 0 & \mathrm{for}\;r>\sqrt[{6}]{2}\sigma \end{array}\right.$$

where *r* is the distance between two monomers, and σ and ε are the length parameter and the interaction strength, respectively. It is a purely repulsive potential and can be used to simulate polymers in good solvent. A quadratic potential is used to model bonding between monomers, given by

(7) $$U_{\mathrm{bd}}\left(b\right)=\frac{1}{2}k{\left(b-b_{0}\right)}^{2}$$

where *b* and $b_{0}$ are the actual and the equilibrium bond lengths, respectively, and *k* is the spring constant. The mass of a monomer is *m* and the thermal energy is $k_{B}T$. The two quantities, together with σ, are chosen to be the mass, the energy, and the length units of the simulation, respectively. We set $\varepsilon =1.2\;k_{B}T$, $b_{0}=1.0\;\sigma$, and $k=6000\;\frac{k_{B}T}{\sigma^{2}}$.

The usage of the bead-spring chain model allows us to investigate properly how the expansion behaviors are derived from a simple connected chain structure. The WCA potential is chosen so that the beads have an effective hard-sphere diameter of about σ against the thermal energy. The spring constant is chosen, corresponding to the order of a stretching force constant of chemical bond. We have verified that *k* is strong enough to maintain the bond length around $b_{0}$ with the fluctuation of bond smaller than 1%. Therefore, crossing between chain segments is not possible in the simulations. Langevin thermostat is used to control temperature at *T* and simulate thermal fluctuations. The friction coefficient η is set to be $20.0\;\frac{\sqrt{mk_{B}T}}{\sigma }$, which imitates an aqueous environment. The equations of motion are solved numerically by using LAMMPS [71] with the integration time step $\Delta t=0.005\;\sigma \sqrt{\frac{m}{k_{B}T}}$.

**Figure 1:**
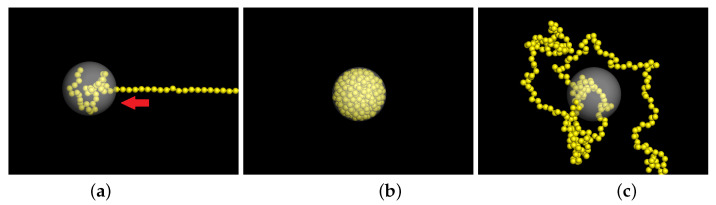
Three phases of simulation: (**a**) the loading phase, in which a chain is loaded into the cavity via a small open pore, (**b**) the equilibrating phase, in which the chain is equilibrated inside the cavity, and (**c**) the releasing phase where the chain is released by switching off the reflection interaction of the cavity wall. The yellow beads represent the monomers of chain. The white sphere represents the cavity. The chain length *N* is 256 and $\phi_{0}$ is 0.4.

The chain is initially confined in a spherical cavity. The beads and the cavity wall interact via a reflection interaction by setting the bead radius to 0.5σ. At a given volume fraction of confinement $\phi_{0}$, the cavity has the diameter equal to $D=\sigma {\left(\frac{N}{\phi_{0}}\right)}^{1/3}$. The simulation comprises three phases. The first phase is the loading phase in which the chain is loaded into the cavity by a pumping force set on a small pore opened on the wall. The second is the equilibrating phase where the confined chain is equilibrated in the cavity by running $10^{8}$ time steps. The third one is the releasing phase: we switch off the reflection interaction between the beads and the wall, and the chain is released and expands in the solution. Several physical quantities are calculated and monitored during the process to study the expanding kinetics. Snapshots of simulation in the three phases are presented in Figure 1 for the case N=256 and $\phi_{0}=0.4$.

We vary systematically the confining volume fraction from $\phi_{0}=0.4$ down to 0.05. The chain length is varied from N=32 to 512. For each studied case (*N*, $\phi_{0}$), 1000 independent runs are performed. The data are recorded for statistical analysis.

In the following text, the simulation units, *m*, $k_{B}T$, σ, will not be shown in the reporting results in order to reduce the notation. For example, a reported time t=2.5 means that *t* is equal to $2.5\;t_{u}$ where $t_{u}=\sigma {\left(\frac{m}{k_{B}T}\right)}^{1/2}$ is the simulation time unit. To map the simulation data to a real system such as single-strand DNA or RNA chains, we can choose σ to be 3.4 Å, m=320g/mol, and $k_{B}T=300k_{B}=4.14\times 10^{-21}\;J$. The simulation time unit $t_{u}$ is thus equal to 3.86ps.

## 4. Results

### 4.1. Chain Size versus Time during an Expansion

**Figure 2:**
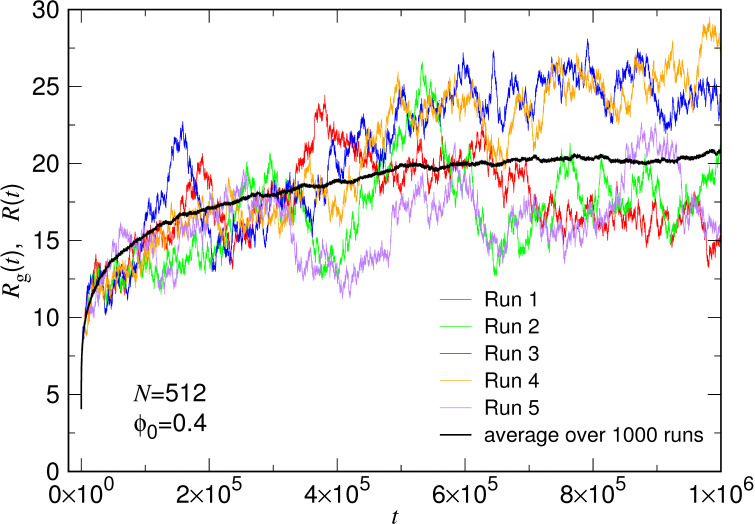
Variations of chain size $R_{g}\left(t\right)$ in five single runs for the case $\left(N,\phi_{0}\right)=\left(512,0.4\right)$, denoted by Run 1 to Run 5 in the legend. The averaged variation R(t) over 1000 independent runs is plotted in black color for comparison.

In this work, we study the variation of chain size by calculating the radius of gyration of chain during an expansion, given by the formula

(8) $$R_{g}\left(t\right)={\left[\frac{1}{N}\sum_{i=1}^{N}{\left(r_{i}\left(t\right)-r_{\mathrm{cm}}\left(t\right)\right)}^{2}\right]}^{1/2}$$

where $r_{i}\left(t\right)$ is the position of a monomer *i* at time *t* and $r_{\mathrm{cm}}\left(t\right)$ is the center of mass of the chain. Figure 2 presents the calculated $R_{g}\left(t\right)$ curves in five single runs for a system of the chain length N=512, released from a confining volume fraction $\phi_{0}=0.4$. The release starts with an initial chain size equal to 4.05(3) at t=0, and the size increases with time in a zigzagged way toward a final value ranged between 15 and 28. The large zigzag shows that the passage of chain expansion suffers from strong thermal fluctuations. In order to get rid of the fluctuations, 1000 independent runs are performed and the averaged chain size at time *t* is computed by the following way:

(9) $$R\left(t\right)={\langle R_{g}^{2}\left(t\right)\rangle }^{1/2}.$$

The result has been plotted in the figure, in black-colored line for comparison. The averaged curve exhibits smooth growing behavior, allowed for further analysis. It shows that a large number of sampling is necessary for the study of the kinetic behavior of polymer expansion.

**Figure 3:**
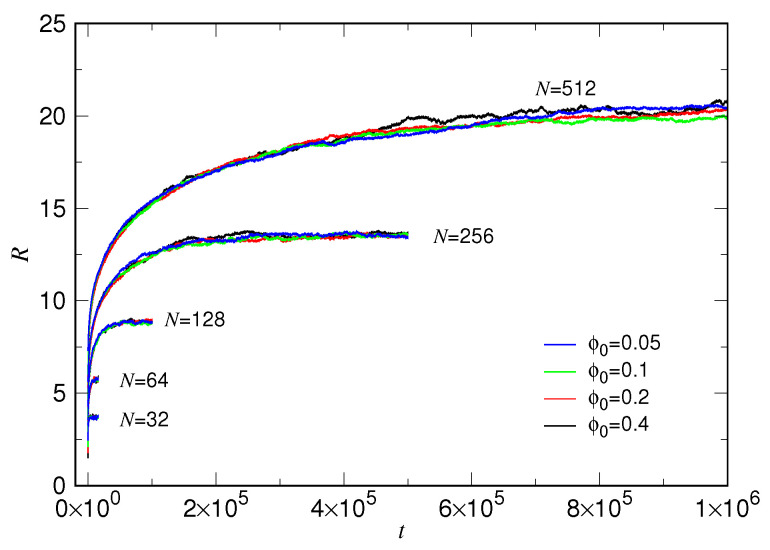
Averaged variation of chain size R(t), for chain length N=32, 64, 128, 256, and 512 released from $\phi_{0}=0.4$, 0.2, 0.1, and 0.05. The chain length is indicated near the corresponding group of the curves, while the volume fraction is given in the legend.

In order to have a thorough picture of the expansion behaviors, we performed simulations for the systems with $N=2^{g_{N}}$ released from the $\phi_{0}=0.4\times 2^{-g_{F}}$ condition, by varying $g_{N}$ from 5 to 9 and $g_{F}$ from 0 to 3. The averaged variations of chain size for the various conditions are presented in Figure 3, plotted in a linear time scale to have a global view.

We, astonishingly, found that the R(t) curves are separated into groups, depending on the chain length. The dependence on the initial volume fraction $\phi_{0}$ is weak in each group, barely seen in the figure. We see also that the expansion duration spans several orders in time for different chain lengths.

**Figure 4:**
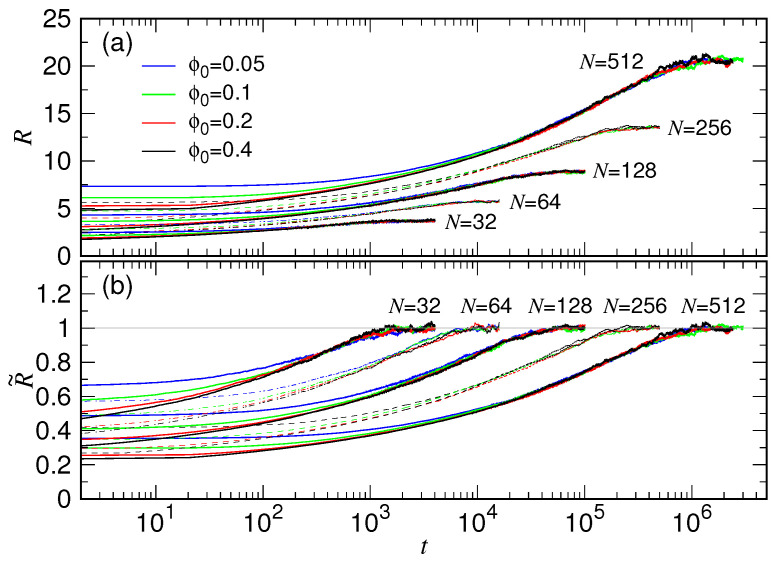
(**a**) R(t) vs. *t* plotted by using logarithmic scale for the time axis. (**b**) Normalized chain size $\tilde{R}\equiv R/R_{F}$ vs. *t* in the semi-log plot. The chain length *N* is indicated near the group curves. The legend for $\phi_{0}$ can be read in Panel (**a**) of the figure.

To see clearly the spanning behaviors and the dependence on $\phi_{0}$, we replot the expansion curves in Figure 4a by using log scale. The $\phi_{0}$-dependence in each group curves appears only in the beginning of expansion (i.e., in the small *t* regime). By tracing in the reverse time direction, a group curve separates into individual ones, each tends toward the initial chain size given by the confining condition $\phi_{0}$.

**Figure 5:**
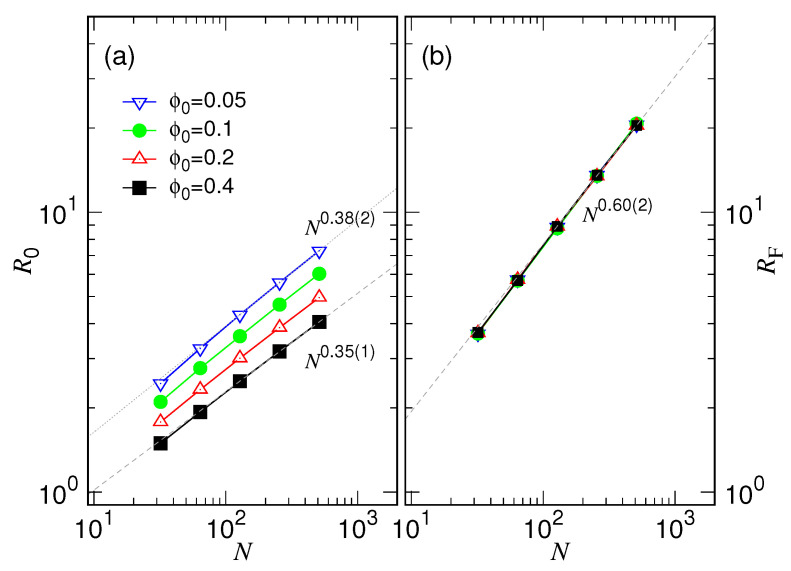
(**a**) The initial chain size $R_{0}$ vs. *N* and (**b**) the final chain size $R_{F}$ vs. *N* in the log–log plot. The value of $\phi_{0}$ is given in the legend of Panel (**a**).

We have studied the initial chain size $R_{0}$ at t=0 and the final size $R_{F}$ when the R(t) curve reaches a steady value. The results are given in Figure 5.

We can see that $R_{0}$ scales like $N^{0.35(1)}$ at $\phi_{0}=0.4$, very close to the expected $N^{\frac{1}{3}}$ behavior, because the initial chain size should be proportional to the cavity diameter *D* through the relation $D=\sigma {\left(\frac{N}{\phi_{0}}\right)}^{\frac{1}{3}}$. However, as $\phi_{0}$ decreases, we observed that the scaling exponent increases slightly, becoming 0.38(2) for the case at $\phi_{0}=0.05$. The deviation comes from the fact that the distribution of the monomers in the cavity is not perfectly uniform, and, thus, $R_{0}$ is not truly proportional to *D*. The non-uniform effect augments as $\phi_{0}$ decreases because in a larger cavity, the confined chain has an increased chance to be not a good sphere. We will show it later. Concerning the relaxed chain size, we obtained the well-known scaling behavior $R_{F}∼N^{0.60(2)}$ in Panel (b), which is independent of $\phi_{0}$.

We then compare the expansion behavior over different chain lengths. The chain size R(t) is normalized by the final size $R_{F}$, as having been plotted in Figure 4b. Now we see clearly how the separated curves vary from their initial values and converge towards the final grouped one. The group curves for different *N* appear in a regular way on the plot, suggesting the existence of some kind of scaling law in the time space.

### 4.2. Scaling Behavior of Chain Size in the Second Stage

**Figure 6:**
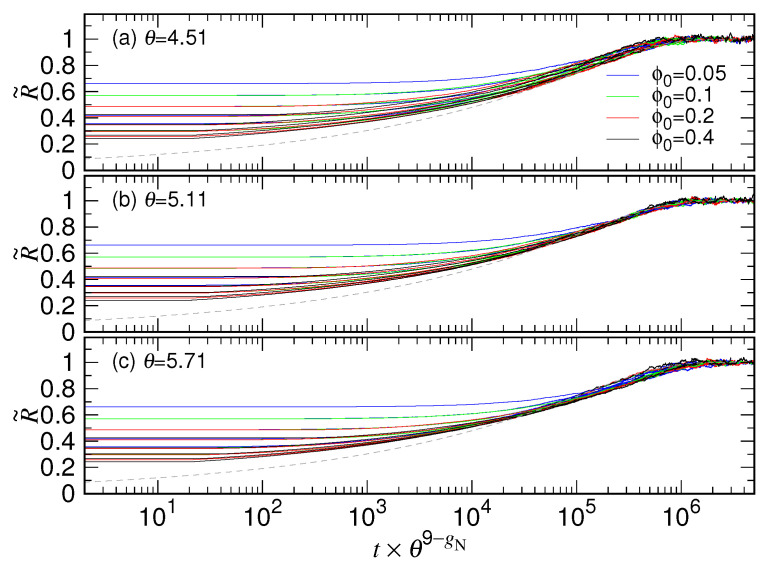
Normalized chain size $\tilde{R}$ vs. the scaled time $t\times \theta^{9-g_{N}}$ plotted for different chain length $N=2^{g_{N}}$ and $\phi_{0}=0.4\times 2^{-g_{F}}$ by using (**a**) θ=4.51, (**b**) θ=5.11, and (**c**) θ=5.71. The confining volume fraction $\phi_{0}$ is given in the legend. The dashed line is the master curve for the second stage, fit from the data in (**b**) in the region with $t\times \theta^{9-g_{N}}>3\times 10^{5}$.

To study the scaling behavior for the variation of chain size, time *t* is scaled by a multiplication factor $\theta^{9-g_{N}}$. We found that by using an appropriate θ, the normalized $\tilde{R}$ curves for different *N* and $\phi_{0}$ can be shifted and collapsed onto a target one in the large time regime (i.e., in the second stage of expansion). Recall that the chain length *N* is chosen as a power of 2 with the exponent denoted by $g_{N}$ in this study. The number 9 appeared in the exponent of the factor states that $N=2^{9}=512$ is the target curve. Figure 6 presents the calculations by using three θ values: 4.51, 5.11, and 5.71.

We observed that with θ=4.51, the shift for the curves on the time axis is somewhat insufficient for the small *N* systems, while the shift is a little bit too much if θ is taken the value 5.71. The ensemble of the scaled curves in the both cases exhibits a broader distribution, particularly seen on the “neck region” of the curves near the scaled time $3\times 10^{5}$. The best collapse occurs at θ=5.11, which produces the finest neck for the ensemble of the curves. The details how we obtained the optimized scaling factor θ with a precision to the second decimal are explained in Section S1 of Supplementary Materials.

Because the initial size $R_{0}$ scales about as $N^{\frac{1}{3}}$ and the final size $R_{F}$ as $N^{0.6}$, the normalized $\tilde{R}$ should attain the value zero at t=0 as *N* tends to infinity. As a consequence, the parameter $b_{c}$ in Equation (5) should be 1, which defines a master curve for the expansion of a chain in the second stage. The master curve can be obtained by fitting the data in the region $t\times \theta^{9-g_{N}}>3\times 10^{5}$ in (Figure 6b), and has been plotted in the figure in dashed line for reference.

**Figure 7:**
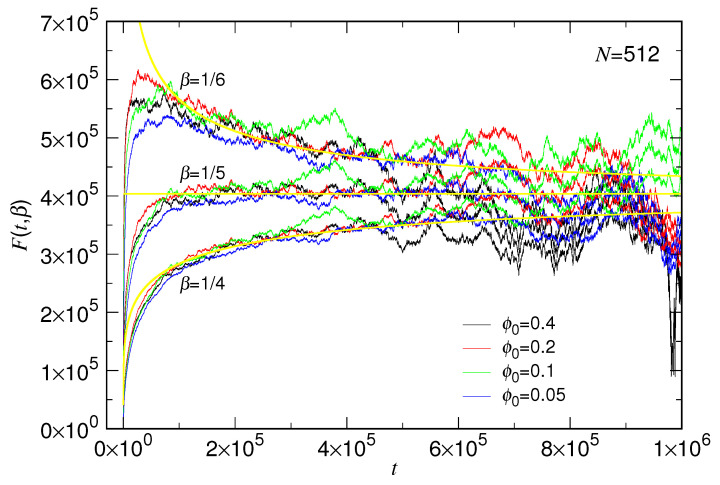
Quantity $F\left(t,\beta \right)=-t/\ln\left(1-{\tilde{R}}^{\frac{1}{\beta }}\left(t\right)\right)$ calculated from the simulation data for N=512, plotted against *t* at $\beta =\frac{1}{4}$, $\frac{1}{5}$ and $\frac{1}{6}$. The value of $\phi_{0}$ is given in the legend. The theoretical curves for F(t,β) are plotted in yellow color by assuming $\tilde{R}\left(t\right)={\left(1-\exp\left(-\frac{t}{4.03\times 10^{5}}\right)\right)}^{1/5}$.

Concerning the characteristic time $\tau_{c}$, we verify first that the variation of chain size in the second stage can be described by the equation

(10) $$\tilde{R}\left(t\right)={\left(1-\exp\left(-\frac{t}{\tau_{c}}\right)\right)}^{\beta }$$

with $\beta =\frac{1}{5}$. The quantity $F\left(t,\beta \right)=-t/\ln\left(1-{\tilde{R}}^{\frac{1}{\beta }}\left(t\right)\right)$ was calculated from the simulation data for N=512 by using three β values, $\frac{1}{4}$, $\frac{1}{5}$ and $\frac{1}{6}$, and the results are presented in Figure 7. A good choice of β shall produce a constant quantity against time and the constant value is $\tau_{c}$.

One can see that $\beta =\frac{1}{5}$ gives the best, leveled-off curves at a value around $4.03\times 10^{5}$ in the time regime $t>10^{5}$ for the four $\phi_{0}$ cases, while the curves increase with time by choosing $\beta =\frac{1}{4}$ and decrease by using $\beta =\frac{1}{6}$. Moreover, using the obtained $\tau_{c}$ value, we are able to calculate theoretical F(t,β) by assuming $\tilde{R}\left(t\right)={\left(1-\exp\left(-\frac{t}{\tau_{c}}\right)\right)}^{1/5}$. The theoretical curves for the three studied β values have been plotted in the figure in yellow line. We can see that the variation trends of the simulation data with respect to the different β can be well captured by the yellow curves. The results suggest strongly $\beta =\frac{1}{5}$. Large fluctuations exhibit later when *t* is larger than $5\times 10^{5}$. It is because $\tilde{R}$ approaches 1 and, thus, the denominator of F(t,β) becomes divergent. Consequently, the uncertainty of $\tilde{R}$ leads to the big fluctuations.

We have calculated another quantity $G\left(t,\tau_{c}\right)=\ln\left(1-\exp\left(-\frac{t}{\tau_{c}}\right)\right)/\ln\tilde{R}\left(t\right)$ for N=512 by assuming $\tau_{c}=4.03\times 10^{5}$. This quantity is expected to attain the value $\beta^{-1}$, allowing a direct verification of the exponent. The results are presented in Supplementary Materials, Figure S3. We observed that $G(t,\tau_{c})$ evolves to a constant value around 5, distinguishable from 4 and 6. It supports again that β is $\frac{1}{5}$.

**Figure 8:**
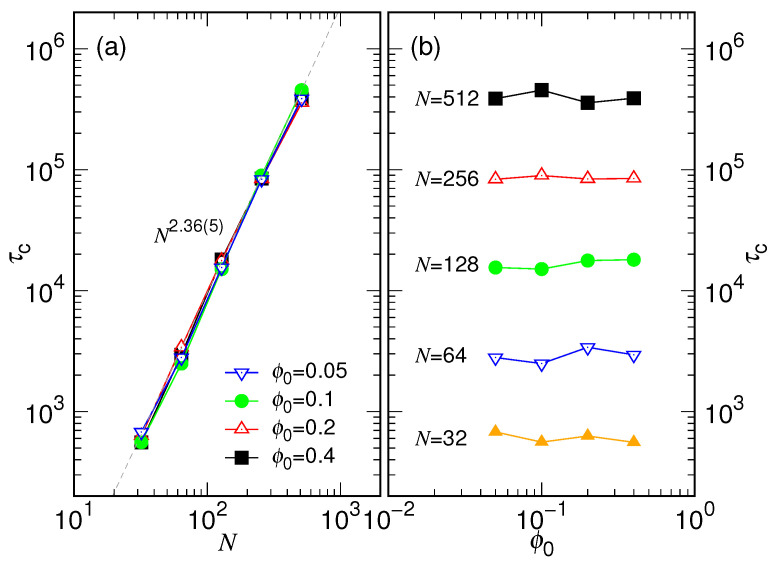
(**a**) $\tau_{c}$ vs. *N* for a given $\phi_{0}$. The value of $\phi_{0}$ can be read in the legend. (**b**) $\tau_{c}$ vs. $\phi_{0}$ at a fixed *N*. The chain length *N* is indicated near the corresponding curve.

Once knowing the β value, we can now determine $\tau_{c}$ via a single-parameter fit of Equation (10) in the large time regime. The obtained $\tau_{c}$ values for different releasing conditions are given in Figure 8, plotted against *N* in Panel (a) and against $\phi_{0}$ in Panel (b).

We found that $\tau_{c}$ scales as $N^{x_{c}}$ with $x_{c}=2.36\left(5\right)$ and is independent to the varying of $\phi_{0}$. The obtained exponent $x_{c}$ corresponds to a θ-value equal to $2^{2.36}≃5.13$, which agrees with the optimal θ used in collapsing the variational curves in Figure 6.

**Figure 9:**
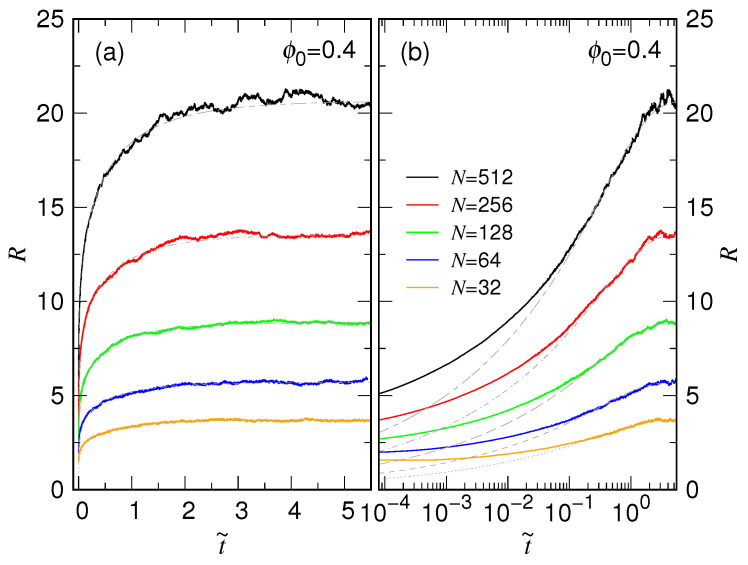
Fitting chain expansion in the second stage for $\phi_{0}=0.4$. The scaled time $\tilde{t}=t/\tau_{c}$ is used to make the plot. Panel (**a**) is plotted in linear scale of $\tilde{t}$ while Panel (**b**) is plotted in logarithmic scale. The chain length can be read in the legend.

Figure 9 presents the results of fitting for $\tau_{c}$ at $\phi_{0}=0.4$. Because the relaxation time has several orders of difference for different chain lengths, we use the scaled time $\tilde{t}=t/\tau_{c}$ to make the plot in order to see clearly the fitting curves.

Panel (a) is the plot in linear scale while Panel (b) is a replot by using the logarithmic scale. The fitting was performed in the large time regime $\tilde{t}>0.3$. A direct comparison of the two panels shows that while looking good globally in Panel (a), the fitting curves deviate from the simulation ones in the small time region, only seen on the log scale in Panel (b).

We notice that the scaling relation $\tau_{c}∼N^{2.36(5)}$ has the exponent $x_{c}$ larger than the predicted one, 2, derived in Section 2. We will discuss about it in Section 5.

### 4.3. Change of Chain Conformation during an Expansion Process

The time parameter $\tau_{c}$ obtained in the previous subsection can serve as a measure to study the progress of an expansion process, owing to the existence of the asymptotic master curve. For example, the total expansion time τ can be defined to be the triple of $\tau_{c}$, the time required for an expanding chain to attain 98% of its final size $R_{F}$ according to Equation (5). Therefore, $\tilde{t}=t/\tau_{c}$ is a dimensionless time which describes the progress of expansion.

**Figure 10:**
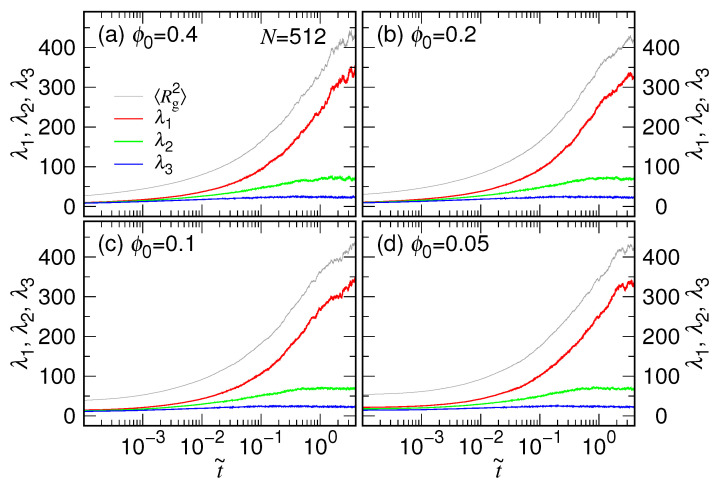
Eigenvalues $\lambda_{1}$, $\lambda_{2}$, $\lambda_{3}$ of [G] as a function of $\tilde{t}$ for N=512, released from (**a**) $\phi_{0}=0.4$, (**b**) $\phi_{0}=0.2$, (**c**) $\phi_{0}=0.1$, and (**d**) $\phi_{0}=0.05$. The $\langle R_{g}^{2}\rangle$ curve is plotted in each panel for comparison. The color representations of the curves are given in Panel (**a**).

Here, we study the change of chain conformation using $\tilde{t}$. The 9 elements of the gyration tensor [G] of chain were calculated via the formula

(11) $$G_{ab}=\frac{1}{N}\sum_{i=1}^{N}\left(r_{i,a}-r_{\mathrm{cm},a}\right)\left(r_{i,b}-r_{\mathrm{cm},b}\right)$$

where *a* and *b* denote ones of the *x*, *y*, or *z* components. The eigenvalues $\lambda_{1}$, $\lambda_{2}$, and $\lambda_{3}$ of [G] were then computed during the process and arranged to be $\lambda_{1}\geq \lambda_{2}\geq \lambda_{3}$. How the eigenvalues vary with the scaled time $\tilde{t}$ is presented in Figure 10 for N=512.

We can see that $\lambda_{1}$, $\lambda_{2}$, $\lambda_{3}$ are identical at the beginning when $\phi_{0}$ is large, such as $\phi_{0}=0.4$ and 0.2. The value trifurcates with passing of the time, and $\lambda_{1}$ becomes much larger than the other two eigenvalues. On the contrary, if $\phi_{0}$ is small, the three eigenvalues have shown differences since the beginning, even though the confining cavity is spherical. For example, we have $\lambda_{1}=21.1\left(3\right)$, $\lambda_{2}=17.6\left(2\right)$, $\lambda_{3}=14.4\left(2\right)$ for the case $\phi_{0}=0.05$ at $\tilde{t}=0$. The distribution of monomers is hence not uniform in the cavity under such a loose confining condition and possesses a structure with unequal mean square distance to the cavity center in the three principal directions. It explains the observed scaling behavior $R_{0}∼N^{\frac{1}{3}+\delta }$ in Figure 5a with a deviated exponent δ. The final eigenvalues are 332.9(9.3), 68.6(7.9), and 22.8(3.5) as time tends to infinity. We have verified that the equality $R^{2}\equiv \langle R_{g}^{2}\rangle =\lambda_{1}+\lambda_{2}+\lambda_{3}$, is valid at any time moment.

The three eigenvalues were then used to compute the two shape factors, called asphericity *A* and prolateness *P* [72], defined by

(12) $$\begin{array}{ccc} A & = & \frac{3(\lambda_{1}^{2}+\lambda_{2}^{2}+\lambda_{3}^{2})}{2{\left(\lambda_{1}+\lambda_{2}+\lambda_{3}\right)}^{2}}-\frac{1}{2} \end{array}$$

(13) $$\begin{array}{ccc} P & = & \frac{27\left(\lambda_{1}-\bar{\lambda }\right)\left(\lambda_{2}-\bar{\lambda }\right)\left(\lambda_{3}-\bar{\lambda }\right)}{2\lambda_{1}^{3}} \end{array}$$

where $\bar{\lambda }$ is the mean value of the three eigenvalues. The asphericity *A* quantifies the degree of the chain shape deviating from a sphere. The value lies between 0 and 1: zero for a perfect sphere and 1 for a rod structure. The prolateness *P* is used to distinguish a prolate conformation (P>0) from an oblate one (P<0). The prolate conformation is an elongated configuration occurred when $\lambda_{1}\gg \lambda_{2}≳\lambda_{3}$, whereas the oblate one is a flattened structure with $\lambda_{1}≳\lambda_{2}\gg \lambda_{3}$. Here, we have modified the definition for the prolateness, so that the value of *P* lies between −1 and 1. The conventional definition is $P_{c}=\left(\lambda_{1}-\bar{\lambda }\right)\left(\lambda_{2}-\bar{\lambda }\right)\left(\lambda_{3}-\bar{\lambda }\right)/{\bar{\lambda }}^{3}$, which produces a value between −0.25 and 2, not equal in the ranges for an oblate and a prolate structure [72,73].

**Figure 11:**
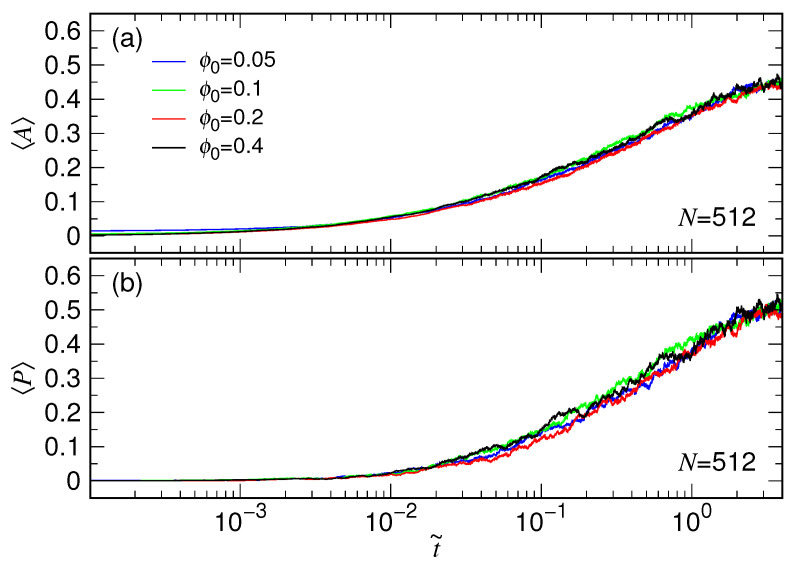
(**a**) Asphericity $\langle A\rangle$ and (**b**) prolateness $\langle P\rangle$, plotted against the scaled time $\tilde{t}$. The chain has N=512, expanding from four different initial volume fraction $\phi_{0}$. The value of $\phi_{0}$ can be read in the legend of Panel (**a**).

Figure 11 presents the averaged $\langle A\rangle$ and $\langle P\rangle$ for the chain N=512 expanding from the four studied $\phi_{0}$ situations.

The curves increase very rapidly so that the logarithmic scale is used to see the variations. Panel (a) shows that the chain deviates from a sphere-like structure in a very short time period, at about $\tilde{t}=0.005$, after the releasing. The $\langle P\rangle$ curves (in Panel (b)) departs from zero and increase also quickly with time. The result follows our anticipation that the expanding chain should become prolate eventually and displays a coil-like structure. We have studied the data in the region $\tilde{t}>3$ and calculated the averaged values. The obtained result is 0.438(7) for $\langle A\rangle$ and 0.497(12) for $\langle P\rangle$. We have also calculated the conventional prolateness $\langle P_{c}\rangle$ and the final value is 0.551(13). These values are consistent with the ones, $\langle A\rangle =0.431$ and $\langle P_{c}\rangle =0.541$, for coil chains reported in literature [73,74].

A notable finding in our study states that the change of chain conformation in an expansion process is not sensitive to the confining volume fraction and follows basically a master curve of evolution against the scaled time $\tilde{t}$.

### 4.4. Scaling Behavior in the First Stage

**Figure 12:**
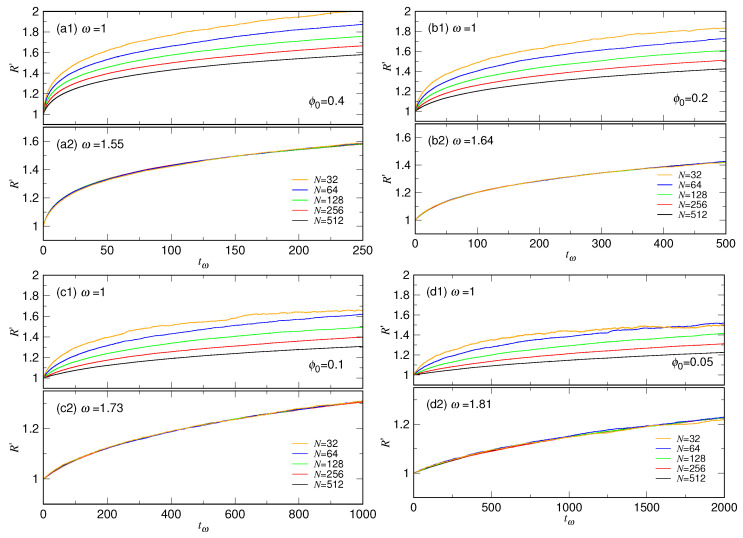
Scaled chain size $R^{\prime }=R/R_{0}$ vs. scaled time $t_{\omega }=t\times \omega^{9-g_{N}}$ for the four studied $\phi_{0}$ cases. The chain length *N* can be read from the legends in (**a2**–**d2**). Panels (**a1**–**d1**) are plotted by setting ω=1. Under the setting, $t_{\omega }$ is just *t* and, thus, the plots present the evolution of $R^{\prime }$ against the ordinary time *t*. Panels (**a2**–**d2**) show the collapse of the time-scaled curves by using the appropriate ω value, 1.55, 1.64, 1.73, and 1.81, respectively.

We have shown that an expanding chain changes its conformation very quickly and maintains the initial sphere-like structure only for a short time, measured by the time scale $\tau_{c}$. To understand further the behavior in the very beginning, we calculate now the scaled chain size $R^{\prime }$, defined to be *R* divided by the initial chain size $R_{0}$. The results, $R^{\prime }$ vs. *t*, are plotted in Figure 12 in linear scale, categorized by the four studied $\phi_{0}$ in Panel (a1), (b1), (c1), and (d1), respectively.

We can see that the $R^{\prime }$ curves for different *N* depart from the value 1 and increase in a similar way in each panel. We found that by multiplying a suitable scaling factor $\omega^{9-g_{N}}$ to the time *t*, similar to the analysis performed in Figure 6, the curves can be stretched and collapsed onto the one for N=512. The results are given in Panel (a2), (b2), (c2), and (d2). The optimal ω value for the best collapse is 1.55, 1.64, 1.73, and 1.81, respectively, for $\phi_{0}=0.4$, 0.2, 0.1, and 0.05. Readers can refer to Supplementary Materials, Section S3, for the explanation how we obtained the values.

**Figure 13:**
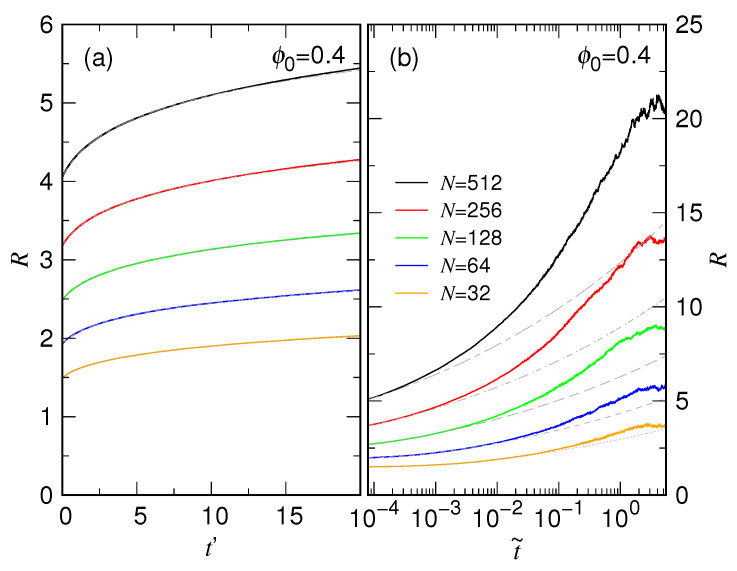
Fitting of the chain size in the first stage of expansion by using the two-parameter fit of Equation (3). The $\phi_{0}$ is 0.4 and the chain length *N* is indicated in the legend. The fitting curves are plotted in gray dashed lines. Panel (**a**) is plotted by using the scaled time $t^{\prime }=t/\tau_{s}$ in linear scale, for the purpose to check the validity of the fitting. Panel (**b**) is plotted by using $\tilde{t}=t/\tau_{c}$ in logarithmic scale, to have a global view of the fitting curves in the expansion process.

The collapse of the curves reveals an important scaling property under the fixed-$\phi_{0}$ condition: the chain does expand like a sphere for a while; however, the duration is quite short in a time span measured by some characteristic time, which should be $\tau_{s}$. We therefore fit the very-beginning behavior of *R* by using Equation (3) by considering $\tau_{s}$ and α as two fitting parameters. The fitting was performed iteratively by using Levenberg–Marquardt algorithm in the time range $\left[0,10\tau_{s}\right]$, until the convergence of $\tau_{s}$. A demonstration of the fitting results is given in Figure 13a for the case $\phi_{0}=0.4$, plotted with the scaled time $t^{\prime }=t/\tau_{s}$.

We see that the simulation data can be well described by the fitting curves, showing the validity of using Equation (3) to approximate the size expansion in the first stage. Figure 13b shows how the fitting curves look like in the whole expansion process, plotted by using the other scaled time $\tilde{t}=t/\tau_{c}$ in logarithmic scale. It is clear that Equation (3) cannot describe the chain size in the second stage.

**Figure 14:**
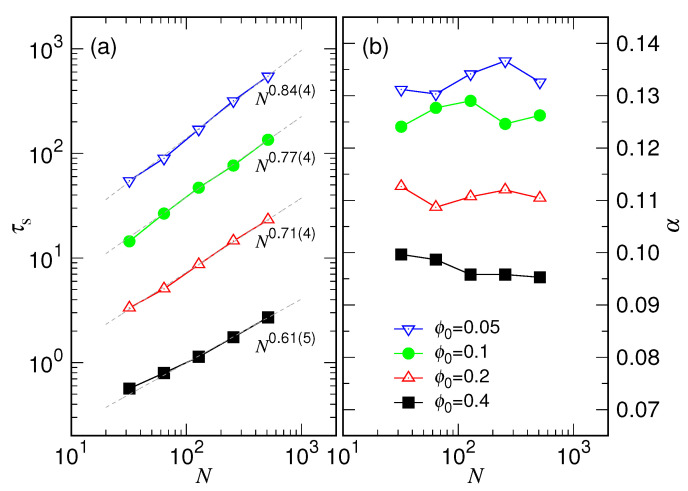
(**a**) $\tau_{s}$ vs. *N* and (**b**) α vs. *N* for different $\phi_{0}$. The value of $\phi_{0}$ can be read in the legend of Panel (**b**).

The obtained parameters $\tau_{s}$ and α are plotted against *N* in Figure 14 for scaling analysis.

We found that $\tau_{s}$ follows a power law $N^{x_{s}}$ with the exponent $x_{s}$ equal to 0.61(5), 0.71(4), 0.77(4), and 0.84(4), respectively, for the four $\phi_{0}$ cases in Panel (a). Because $\tau_{s}$ is the scaling time for the chain variation in the first stage, the exponent $x_{s}$ should relate to the scaling factor ω used for the collapse of the $R^{\prime }$ curves in Figure 12 by the way: $x_{s}=\log_{2}\omega$. The four obtained ω values give the corresponding $x_{s}$ equal to 0.63, 0.71, 0.79, and 0.86, respectively, which are in good agreement with the results of the scaling analysis.

Figure 14b shows that the exponent α is roughly constant at a given $\phi_{0}$ with respect to varying of the chain length. To check the consistency, we calculated the quantity $H\left(t,\tau_{s}\right)=\log\left(R^{\prime }\right)/\log\left(1+\frac{t}{\tau s}\right)$ by using the $\tau_{s}$ value obtained in Figure 14a. The quantity is expected to attain the value α in the first stage.

**Figure 15:**
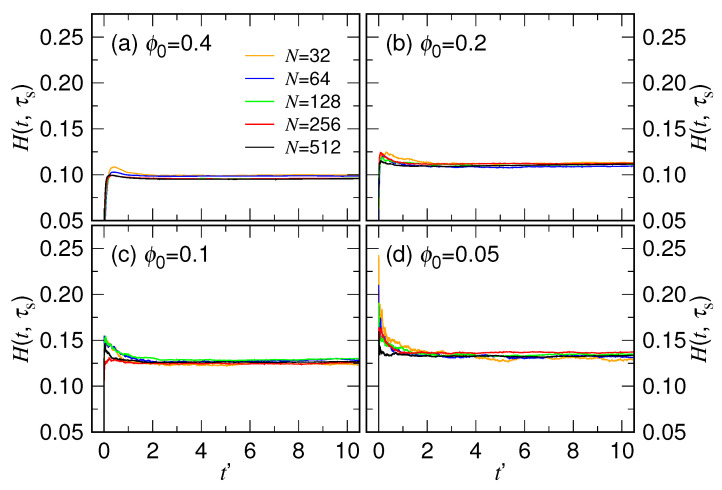
Quantity $H\left(t,\tau_{s}\right)=\log\left(R^{\prime }\right)/\log\left(1+\frac{t}{\tau_{s}}\right)$ calculated by using the simulation data $R^{\prime }$ and the fitting parameter $\tau_{s}$, plotted against $t^{\prime }$ for the case (**a**) $\phi_{0}=0.4$, (**b**) $\phi_{0}=0.2$, (**c**) $\phi_{0}=0.1$, and (**d**) $\phi_{0}=0.05$. The chain length can be read in the legend of Panel (**a**).

We can see in Figure 15 that $H(t,\tau_{s})$ is basically constant in the fitting region except in the very beginning $t^{\prime }≲1$, where $H(t,\tau_{s})$ departs from zero at $t^{\prime }=0$, exhibits a peak, and then evolves to become a plateau. The plateau for the four studied cases has a value of about 0.980, 0.108, 0.126, and 0.133, respectively, corresponding well to the α value obtained by fitting. The deviation of *H* from a plateau near $t^{\prime }=0$ indicates that the size variation does not truly follow Equation (3) at the starting point of expansion. It is understandable because Equation (3) was derived under a quasi-equilibrium assumption. Therefore, it describes only the steady-state behavior, unable to depict the transient one just after the releasing of the chain from a cavity. We will discuss more about it later.

According to our scaling theory, the exponent α is predicated $\frac{3\nu_{b}-1}{6\nu_{b}+1}$, or inversely, the scaling exponent $\nu_{b}$ can be calculated from α via the equation $\nu_{b}=\frac{1+\alpha }{3-6\alpha }$. It allows us to obtain an estimate for the value of $\nu_{b}$, which is 0.455, 0.471, 0.502, and 0.515, respectively, computed from the four plateau values. Since these $\nu_{b}$’s are all close to 0.5, the ingredients of the blobs constituted in the calculation of the free energy in Equation (2) should be in a melt state. Concerning the characteristic time, the scaling theory predicts $\tau_{s}∼R_{0}^{\frac{1}{\alpha }}N^{\frac{-1}{3\nu_{b}-1}}$. For a general scaling $R_{0}∼N^{\frac{1}{3}+\delta }$ for the initial chain size in a confined cavity, the theory yields $\tau_{s}∼N^{\frac{2}{3}+\frac{\delta }{\alpha }}$. The simulations have given the resulting exponent $x_{s}$ from 0.61(5) to 0.84(4) in Figure 14a, which falls roughly in the same range $x_{s}≳\frac{2}{3}$, compared to the prediction. We are not able to make a conclusive comparison further because it requires a very accurate measurement for both of the exponents δ and α. In particular, the α exponent is relatively small and appears in the denominator, which can easily amplify the error of δ in calculating the predicted $x_{s}$. Additionally, the scaling exponents should be obtained in the limit of infinite chain length. Whether the studied chains have been long enough to approach the limit or be able to reduce the finite-size effect to a considerably small level requires much investment of research, which is beyond the reach of the current study. Nonetheless, the simulations presented here show supporting evidences to the scaling behaviors predicted by the theory.

### 4.5. Speed of Expansion

**Figure 16:**
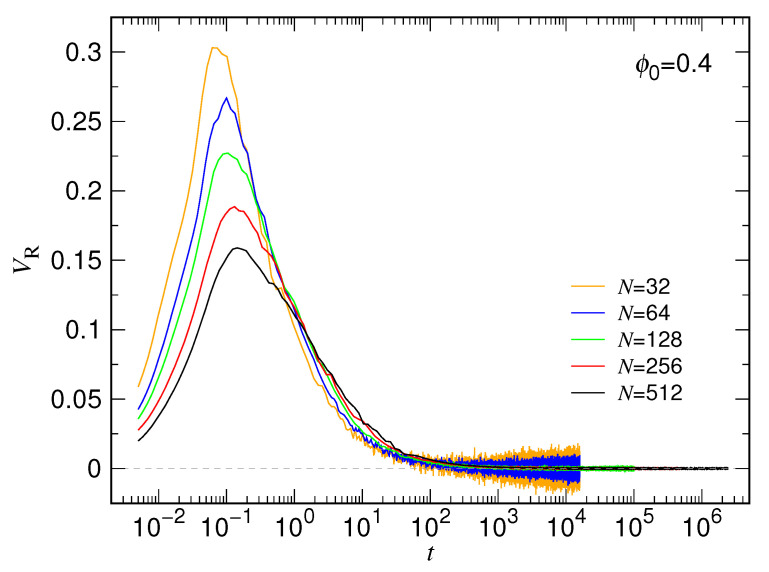
Expansion velocity $V_{R}$ as a function of time *t* for $\phi_{0}=0.4$. The chain length *N* can be read in the legend.

The rate of change of the chain size describes how fast a chain expands during a process. In this subsection, we study of the mean expansion speed $V_{R}=\langle \frac{dR_{g}}{dt}\rangle$, obtained by calculating first the numerical differential of single trajectories of the radius of gyration $R_{g}$ via a three-point formula [75] and then averaging them over the 1000 independent runs. Figure 16 presents the results of calculation for the case $\phi_{0}=0.4$.

Since the chain is initially enclosed in a cavity, $V_{R}$ is zero at t=0. When the expansion starts, the velocity surges, quickly reaches a maximum, and then decreases. The curve begins to show thermal fluctuations when the velocity becomes small to a certain extent. The shorter the chain length, the larger the fluctuations.

Theoretical prediction for the expansion speed can be obtained by taking the time derivative of Equations (3) and (5). The predicted velocities are

(14) $$\begin{array}{ccc} \frac{dR}{dt} & = & \frac{\alpha R_{0}}{\tau_{s}}{\left(1+\frac{t}{\tau_{s}}\right)}^{\alpha -1} \end{array}$$

(15) $$\begin{array}{ccc} \frac{dR}{dt} & = & \frac{\beta R_{F}}{\tau_{c}}{\left(1-\exp\left(-\frac{t}{\tau_{c}}\right)\right)}^{\beta -1}\exp\left(-\frac{t}{\tau_{c}}\right) \end{array}$$

for the two stages. A noticed problem comes from the 1st-stage equation which predicts a non-zero initial expansion velocity of $V_{R}=\frac{\alpha R_{0}}{\tau_{s}}$. This contradicts the fact and is a drawback of the prediction. As mentioned in the previous subsection, the theory is designed to describe the quasi-equilibrium behavior because of the assumption of instant balance between the free energy change and the energy dissipation in the derivation. It cannot depict transient behavior such as how an expanding chain gains its velocity from a starting static state.

**Figure 17:**
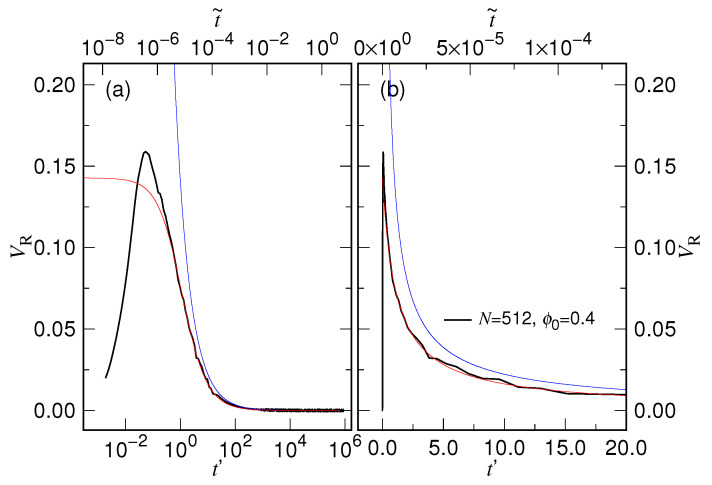
Expansion velocity $V_{R}$ vs. scaled time $t^{\prime }=t/\tau_{s}$ for N=512 with $\phi_{0}=0.4$, plotted in (**a**) logarithmic scale and (**b**) linear scale. The simulation curve is drawn in black color, the velocity calculated from Equation (14) in red color, and the velocity calculated from Equation (15) in blue color. The process described by the scaled time $\tilde{t}=t/\tau_{c}$ can be read on the top of the plots.

Figure 17 compares the simulation results with the two velocity equations by taking N=512 with $\phi_{0}=0.4$.

We can see that the predicted first-stage velocity curve (the red one) deviates from the peaked simulation curve (the black one), exhibiting a plateau in the very beginning of the expansion in the logarithmic plot, Panel (a). It evolves to merge with the simulation curve at $t^{\prime }≃1$. The non-consistency has a duration of about $1\tau_{s}$, which has been seen in Figure 15. The second-stage velocity curve joins the simulation one when $\tilde{t}>10^{-3}$. The scaled time $\tilde{t}=t/\tau_{c}$ has been indicated on the top of the plot to describe the process. Panel (b) presents the variations in linear scale. The velocity surges abruptly and sharply at $t^{\prime }=0$. One can hardly see the difference between the simulation and the predicted first-stage curves.

**Figure 18:**
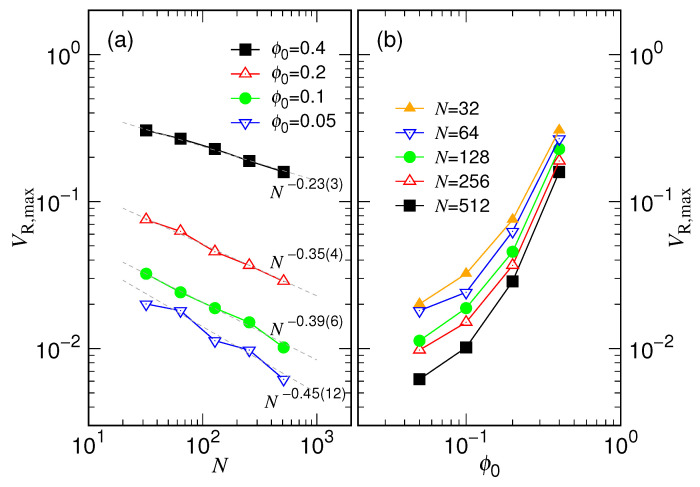
Maximum expansion velocity $V_{R,\max}$ as a function of *N* at a fixed $\phi_{0}$ (Panel (**a**)) and as a function of $\phi_{0}$ for a given *N* (Panel (**b**)). The value of the fixed $\phi_{0}$ in (**a**) and the given *N* in (**b**) can be read in the legend of the panels.

How the maximum velocity $V_{R,\max}$ varies with *N* and $\phi_{0}$ is presented in Figure 18.

We can see that $V_{R,\max}$ exhibits a power-law decrease with *N* under the fixed $\phi_{0}$ condition, as shown in Panel (a). If it is the chain length being fixed, the higher the confining volume fraction, the larger the maximum velocity (refer to Panel (b)). The behavior follows our intuition.

## 5. Conclusions and Discussions

We have developed a two-stage model to describe the expansion process of a chain releasing from a spherical cavity. The kinetic equation of chain size was derived by balancing the rate of the free energy change with the rate of the energy dissipation of the chain during the process. In the first stage, the chain undergoes a fast expansion. We assumed that the chain expands with a spherical conformation and predicted the variation of chain size to be $R\left(t\right)=R_{0}{\left(1+\frac{t}{\tau_{s}}\right)}^{\alpha }$ with $\alpha =\frac{3\nu_{b}-1}{6\nu_{b}+1}$ and $\tau_{s}∼R_{0}^{1/\alpha }N^{\frac{-1}{3\nu_{b}-1}}$. In the second stage, the speed of expansion slows down greatly and the chain is relaxed by maintaining a coil-like structure. It is called the coil expansion and the size variation was predicted to follow $R\left(t\right)=R_{F}{\left(1-\exp\left(-\frac{t}{\tau_{c}}\right)\right)}^{\beta }$ where $\beta =\frac{1}{5}$ and $\tau_{c}∼N^{2}$.

Extensive Langevin dynamics simulations were then performed to verify the theory by varying systematically the chain length *N* and the confining volume fraction $\phi_{0}$. We have shown that the expansion behavior of chain can be well described by the two-stage model. The resulting $\tau_{s}$ scales as $N^{x_{s}}$, and the exponent $x_{s}$ and the exponent α both depend on the $\phi_{0}$, as having been shown in Figure 14. The characteristic time $\tau_{c}$ for the second stage scales such as $N^{x_{c}}$ with $x_{c}$ equal to 2.36(5), and the exponent β is about 1/5 (refer to Figure 7 and Figure 8).

**Figure 19:**
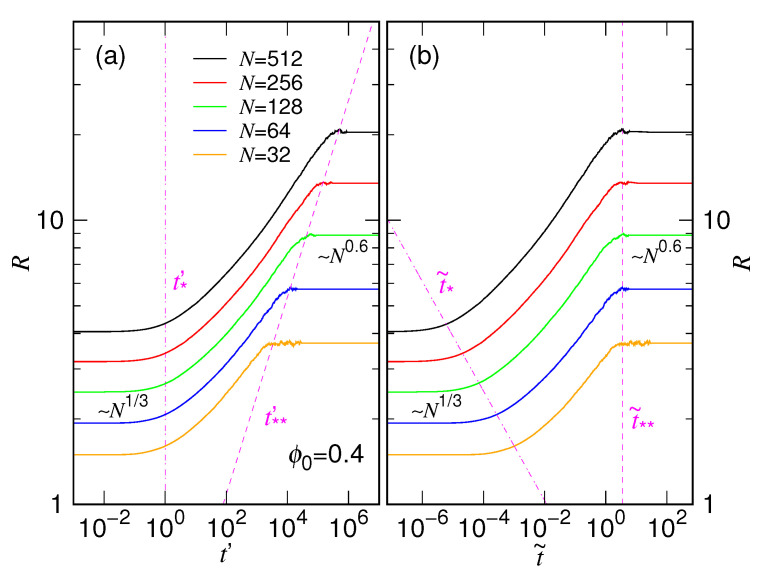
Evolution of the chain size for the case $\phi_{0}=0.4$ plotted by using (**a**) the $t^{\prime }=t/\tau_{s}$ time scale and (**b**) the $\tilde{t}=t/\tau_{c}$ time scale. The chain length can be read in the legend of Panel (**a**). The lines demarcating the regimes for the sphere-like state and the final coil state are plotted in dash-dotted and dashed lines, respectively, both in magenta color.

To make the scaling behaviors more evident, we present here, in Figure 19, the log–log plot of the evolution of chain size for different chain lengths by using the two scaled times, $t^{\prime }=t/\tau_{s}$ and $\tilde{t}=t/\tau_{c}$, for the case $\phi_{0}=0.4$.

In the $t^{\prime }$ scale (Panel (a)), the curves are “aligned” from the left-hand side of the figure, in the regime corresponding to small *t* where the chain is still sphere-like and scales as $N^{\nu_{s}}$ with $\nu_{s}≃1/3$. Because the expanding time depends on the chain length, the final coil chain is reached at different time moment $t_{**}^{\prime }$. One can show that $t_{**}^{\prime }∼R^{\frac{x_{c}-x_{s}}{\nu_{c}}}$ with $\nu_{c}≃0.6$ being the scaling exponent for the coil chains. The $t_{**}^{\prime }$ curve has been drawn on the figure in dashed magenta line. On the right side of the curve, the chains are fully relaxed. Panel (b) is plotted by using $\tilde{t}$ scale. The evolution curves are aligned from the right-hand side at this time, in the regime $\tilde{t}≳3$. Under the scale, the expanding chains leave the sphere-like state at the time moment ${\tilde{t}}_{*}$. The demarcation line ${\tilde{t}}_{*}∼R^{\frac{x_{s}-x_{c}}{\nu_{s}}}$ has been plotted in the figure in magenta dash-dotted line. We now see clearly how the behaviors of chain expansion are scaled with the two characteristic times in a process.

**Figure 20:**
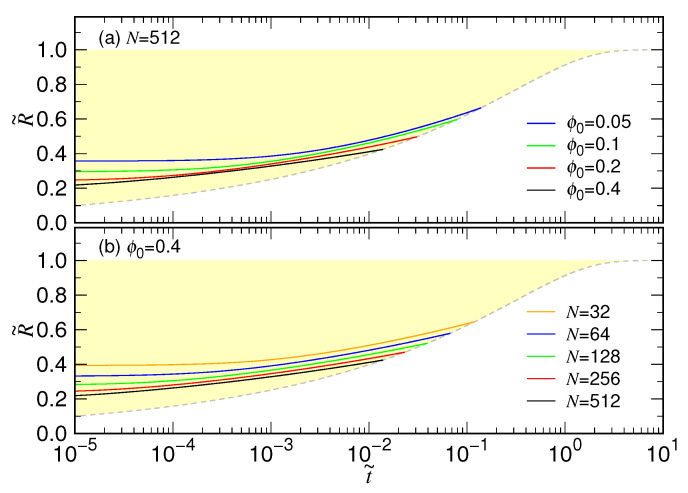
Prediction of chain size plotted by using the scaled time $\tilde{t}$ and length variable $\tilde{R}$. The size variation in the first stage, $\tilde{R}\left(\tilde{t}\right)={\tilde{R}}_{0}{\left(1+\frac{\tilde{t}}{{\tilde{\tau }}_{s}}\right)}^{\alpha }$, are plotted in colored lines (**a**) at N=512 for various $\phi_{0}$ values and (**b**) at $\phi_{0}=0.4$ for various chain lengths. The color representations are given in the legend of each panel. The principal variation curve for the second stage, $\tilde{R}\left(\tilde{t}\right)={\left(1-\exp(-\tilde{t})\right)}^{1/5}$, is plotted in gray dashed line. The yellow region denotes the domain space where situate the size variation curves for the first stage.

Our simulations have revealed that the expansion behavior is determined mainly by the chain length, not sensitive to the confining volume fraction (refer to Figure 3). Because the ratio $\tau_{s}/\tau_{c}$ possesses a quite negative exponent $x_{s}-x_{c}$, the process is largely dominated by the second stage of expansion, particularly when the chain length is long. Therefore, in practice, we see mostly the second-stage behavior and $\tau_{c}$ is a more suitable time scale to describe the expansion. We have defined the expansion time to be $\tau =3\tau_{c}$. Figure 20 presents the theoretical curves by using the scaled time $\tilde{t}$ and the length variable $\tilde{R}=R/R_{F}.$

The gray dashed curve is the principal variation curve for the second stage, defined by $\tilde{R}\left(\tilde{t}\right)={\left(1-\exp(-\tilde{t})\right)}^{1/5}$. The region left to the curve, colored in yellow, is the domain space for the variation of chain size in the first stage, which depends on both *N* and $\phi_{0}$. For a given *N*, a smaller $\phi_{0}$ results in larger $\tilde{R}$ as shown in Figure 20a. If it is $\phi_{0}$ being fixed (see Figure 20b), the larger the chain length, the lower the $\tilde{R}$ curve will be. The variation of chain size turns to follow the universal gray-dashed curve when being in contact with it.

In the study, we have obtained $\tau_{c}∼N^{2.36(5)}$, which is significantly different to the predicted $N^{2}$ behavior. Here we discuss two possible causes which could lead to the difference. The first one comes from the “uniform” setting of the velocities in participation of the energy dissipation in Equation (1) by $\frac{dR}{dt}$. A more precise setting should consider the velocities $v_{i}$ of individual monomers, and the balance equation shall read as

(16) $$\frac{dF}{dt}=-\sum_{i=1}^{N}\eta v_{i}^{2}≃-\int_{V}\eta v^{2}\left(r,t\right)\rho \left(r,t\right)d^{3}r.$$

To make the equation analytic, we can approximate the summation by an integral, but the monomer distribution ρ(r,t) and the velocity distribution v(r,t) at any position **r** in the occupied space V need to be known at any time *t* during the expansion. It will be a very difficult task to obtain these two time-dependent distributions from simulations. However, the non-consistency on the scaling of $\tau_{c}$ can be treated in a different way by modifying the balance equation to be:

(17) $$\frac{dF}{dt}≃-\eta N^{1+\chi }{\left(\frac{dR}{dt}\right)}^{2}$$

with the exponent χ taking into account the additional effect of the monomer and velocity distributions in the integrand and casting it into a power-law dependence on *N*. Our simulations suggest that χ should acquire a value of about 0.36.

The second possible cause is related to the non-equilibrium effect in a process. The expansion of a compressed chain evolves in a very fast way, particularly in the first stage. The balance equation was set under the assumption that the free energy change of chain can be dissipated instantly and completely into the environment. Generally, it is not the case and, therefore, some kind of retardant effect leads to a prolongation of the relaxation, resulting in a larger *N*-exponent for the expansion time. A combination with the first cause yields the effective exponent equal to 2.36(5).

An alternative explanation is to regard the second stage of expansion as a relaxation of a coil chain departing from small deformation, i.e., in a linear response regime. The relaxation time $\tau_{R}$ can be estimated by $\frac{R_{F}^{2}}{D_{R}}$ with $D_{R}$ being the diffusion coefficient of chain in dilute solutions [67]. In Rouse dynamics, $D_{R}$ is known to be $\frac{k_{B}T}{\eta N}$. It yields $\tau_{R}∼N^{1+2\nu_{c}}$; we thus have $\tau_{R}∼N^{2.2}$ by using $\nu_{c}≃0.6$ (refer to Figure 5b). The estimated exponent 2.2 is found close to the one obtained for $\tau_{c}$. The true connection between different kinds of relaxation time of chain and the characteristic time of expansion merits a thorough investigation in the future.

We remark that it has been proposed since the mid-1980s that the collapse of single chains undergoes two-stage kinetics [76,77]. The first stage is a fast crumpling of the chain into a unknotted globule with a required time scale about $\tau_{\mathrm{crum}}∼N^{2}$ and the second one is a slow rearrangement of the chain into a knotted globule via reptations with the estimated time $\tau_{\mathrm{rep}}∼N^{3}$. For a counterpart action, the expansion of chain, we anticipate a shorter time for the disentanglement because the reptation to untie the knots on the chain is biased by entropic forces due to the swelling of the chain itself [41]. Therefore, the scaling exponent for the expansion time should lie between 2 and 3, which agrees with what we have observed here.

Coil-to-globule transition of polymers caused by deterioration of solvent quality has been investigated extensively in the past [78,79,80,81,82,83,84]. Although the expansion study presented here resembles a reverse version of the collapse transition, there exist two main differences:

(1) In the expansion study, it is the external restriction (the confining wall) forcing the chain to be confined in a small space, regardless of the solvent quality, while in a coil-to-globule transition, the globule is formed by internal attractive interactions, which collapses the chain from interior owing to the poor solvent condition. The internal structure of the two kinds of globule are thus different. The globule in the first case can have a less dense center if the intra-chain interaction is repulsive or the chain has stiffness [29,85]. The second case can form so-called “molten globule”, which possesses a dense core with a loosen shell structure [83,86,87]. Consequently, the geometry is less certain, compared to the first one.
(2) The coil-to-globule transition is a relatively slow process. Different intermediate states, such as pearl-necklaces, racquets, folded structures, can be formed in the passage [84,88,89,90,91]. The expansion, on the contrary, is a very fast and violent process. Once the external restriction is removed, the chain expands straightforwardly back to a coil state. No intermediate states are expected if no important attractive interactions are involved. The smaller the confining space, the faster the expansion will be. Even for a globule-to-coil transition induced by improving the solvent quality, the process is not a simple reversal of the coil-to-globule one. Hysteresis has been observed, indicating strong nonequilibrium effects or formation of some kind of intra-chain structures in the process [86].

Therefore, the studied chain expansion should be analogous to the globule-to-coil transition with prudence. Subtle but significant differences appear between them, from the mechanism which drives the transition, to the representation, such as the speed of expansion, and the structures. An in-depth study similar to this is hence necessary to understand how a confined chain expands after releasing from a confinement. Different to the previous globule-to-coil study [41,42,43], we do not observe any clear sign indicating that the released chains are trapped in an arrested state in this study. The chain globules in our confinement hence should not be strongly entangled or tightly knotted. It could simply be a consequence of the lack of local attractive interactions between beads in our simplistic model. Therefore, it is important in the following work to clarify the role of local attractions, such as van der Waals interaction or reversible bond formation [92], on the occurrence of possible arrested states in a chain expansion process.

To have an idea how long it takes for a ssDNA or RNA chain released from a capsid, we can apply the scaling law $\tau_{c}≃0.18N^{2.36}$ obtained in Figure 8. For a chain length of about 1 kbp, the scaling gives $\tau_{c}≃2.16\times 10^{6}$, which is about 8.35μs when converted to the real unit. Therefore, a typical expansion time for releasing ssDNA or RNA from a capsid is of about microsecond order. Previous studies by using detailed simulation models have obtained a similar time order for genome release from an opened capsid [19,20,21]. We emphasize that the results reported here are certainly not quantitative predictions. Biological systems are very complex and have complicated interactions, especially in an aqueous environment where these entities live. Their behaviors are determined by a combination and a competition of different driving forces. Therefore, the simplistic model used here is not possible to give quantitative descriptions. Nonetheless, we have proposed a simple two-stage model to understand free release of a chain from a small cavity: a spherical expansion, followed by a coil expansion. For restricted release happened from an opened capsid, we anticipate more stages in the expansion. For example, the chain globule should be squeezed first to “eject” through the open hole on the capsid, up to a certain extent; it is then followed by a possible “elliptical” expansion combined with the ejection, and finally completed by a coil expansion and diffusion. According to the current study, the characteristic time $\tau_{s}$ can be a thousand times or more smaller than $\tau_{c}$. Therefore, a time resolution of nanosecond order is required to be able to experimentally study the early-stage behavior of genome release. If the chain has stiffness like dsDNA, the expansion time will be shortened further because the stiffness can help restoring the chain back to its coil conformation. To study it, one can include angle potentials in the model to simulate chain stiffness, which will introduce a new length scale to the system, called persistence length. We expect that the expansion behavior will be altered if the cavity diameter becomes comparable to or smaller than the persistence length. Moreover, genetic materials are generally ionizable in solutions. Therefore, it is very important to understand expansion of charged chains in ionic solutions. We will investigate these topics in the future.

## Data Availability

The data presented in this study are available from the corresponding author on reasonable request.

## Supplementary Materials

The following are available online at https://www.mdpi.com/article/10.3390/polym15010198/s1, Figure S1: enlarged plot of Figure 6 to show neck region of the collapsed curves; Figure S2: mean neck width $\langle W_{n}\rangle$ vs. scaling factor θ; Figure S3: quantity $G(t,\tau_{c})$ as a function of *t* for different $\phi_{0}$; Figure S4: mean distribution width $\langle W_{1}\rangle$ vs. scaling factor ω for different $\phi_{0}$.
