# Supplementary material for: Expansion of Single Chains Released from a Spherical Cavity
Source: Polymers (Basel). 2022 Dec 30;15(1):198. doi: 10.3390/polym15010198 (PMC9824584; doi:10.3390/polym15010198)
Supplement: Supplementary file 1 [file polymers-15-00198-s001.zip › polymers-2055415-supplementary.pdf]

Supplementary Information
Expansion of Single Chains Released from a Spherical Cavity

Chia-Cheng Chu[1] and Pai-Yi Hsiao[2,1*]
[1]Institute of Nuclear Engineering and Science, National Tsing Hua University, Hsinchu, Taiwan, R.O.C.
[2]Department of Engineering and System Science,
National Tsing Hua University, Hsinchu, Taiwan, R.O.C.
*Corresponding author, email: pyhsiao@ess.nthu.edu.tw; pyhsiao@mx.nthu.edu.tw

S1. DETERMINATION OF THE θ VALUE FOR COLLAPSING THE $\tilde{R}$ CURVES

In Section 4.2 (Scaling Behavior of Chain Size in the Second Stage), the collapse of the curves of the normalized chain size $\tilde{R}$ in Figure 6 was studied by using different scaling factor θ for time. We observed that the group of the curves exhibits a neck region located in the region $[2 \times 10^5, 4 \times 10^5]$ for the scaled time $t_\theta = t \times \theta^{9-g_N}$. An enlarged plot of Figure 6 is given in Figure S1.

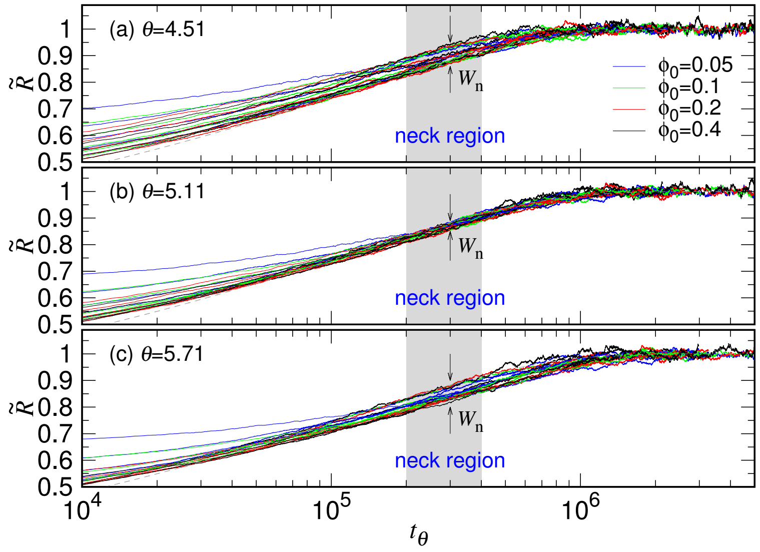

Figure. S1. Replot of Figure $\tilde{R}$ vs. t_θ, in order to see clearly the "neck" of the group of the collapsed curves at the three θ 6, values.

We define the neck width $W_\mathrm{n}(t_\theta)$ to be the difference between the largest and the smallest $\tilde{R}$ in the group curves at a given time point t_θ. Figure S2 presents the results of calculation for the mean width $\langle W_\mathrm{n} \rangle$ in the neck region by scanning the θ value from 4.4 to 6.0 in an increment of 0.01. We can see that the narrowest neck is produced by using $\theta = 5.11$, which represents the best collapse of the curves at a precision of θ to the second decimal.

S2. STUDY OF THE DYNAMIC EXPONENT β FOR THE SECOND STAGE

Here we present a double-check for the value of the dynamic exponent β in the equation

$$\tilde{R}(t) = \left(1 - \exp\left(-\frac{t}{\tau_\mathrm{c}}\right)\right)^{\beta}. \tag{S1}$$

The quantity $G(t, \tau_\mathrm{c}) = \ln\left(1 - \exp\left(-\frac{t}{\tau_\mathrm{c}}\right)\right) / \ln \tilde{R}(t)$ is calculated from the simulation data and plotted against t in Figure S3 for $N = 512$. The parameter τ_c is taken from the level-off value, 4.03×10^5, obtained in Figure 7. We can see that $G(t, \tau_\mathrm{c})$ decreases rapidly and attends a value of about 5 when $t > 10^5$. It agrees well with the prediction $\beta^{-1} = 5$ of our scaling theory. The large fluctuations appeared in the region $t > 5 \times 10^5$ come from the statistical error because the denominator of $G(t, \tau_\mathrm{c})$ tends to zero as t goes to infinity and consequently, the uncertainty is amplified.

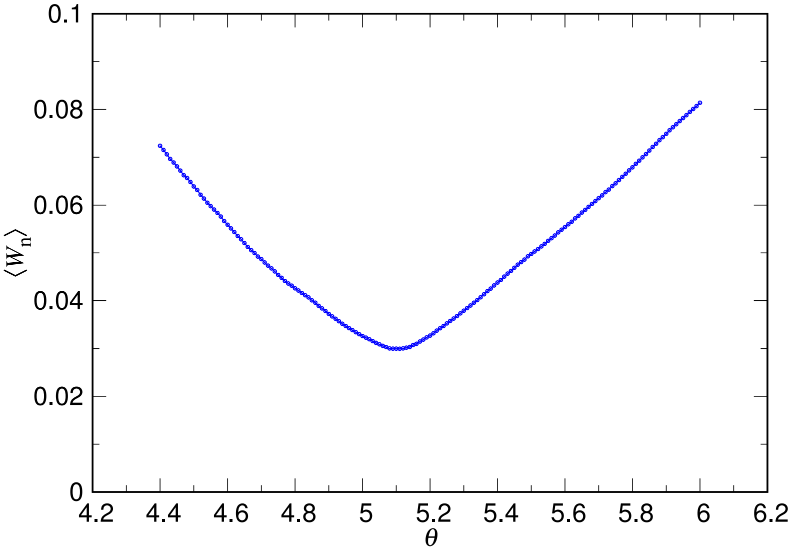

Figure. S2. Mean neck width $\langle W_{\mathrm{n}} \rangle$ of the group curves in the time region $t_\theta \in [2 \times 10^5, 4 \times 10^5]$, as a function of the scaling factor θ.

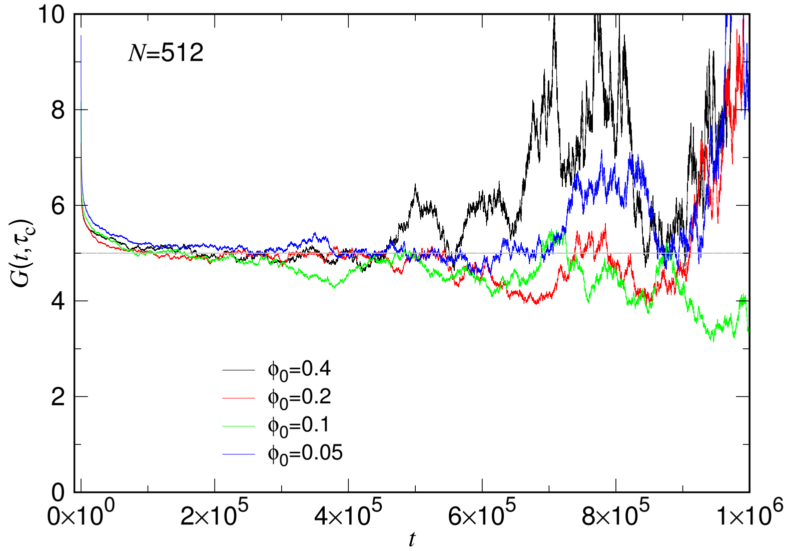

Figure. S3. Quantity $G(t, \tau_{\mathrm{c}}) = \left(1 - \exp\left(-\frac{t}{\tau_{\mathrm{c}}}\right)\right) / \ln \tilde{R}(t)$ calculated for $N = 512$ by setting $\tau_{\mathrm{c}} = 4.03 \times 10^5$. The value of ϕ_0 ln can be read in the legend.

S3. DETERMINATION OF THE ω VALUE FOR BEST COLLAPSE OF THE R' CURVES

In Section 4.4 (Scaling Behavior in the First Stage), the variations of the scaled chain size $R' = R/R_0$ in Figure 12 are collapsed by using an appropriate ω value to scale time through the formula $t_\omega = t \times 2^{9-g_N}$. The way how we determined the ω value is explained below. We calculated the mean distribution width $\langle W_1 \rangle$ for the group of the R' curves in the beginning t_ω region by scanning the ω value from 1.4 to 2.0 in an increment of 0.01. The results, presented in Figure S4, were than used to determine the minimum of $\langle W_1 \rangle$, occurred at $\omega = 1.55$, 1.64, 1.73, and 1.81 for the cases $\phi_0 = 0.4$, 0.2, 0.1, and 0.05, respectively. These ω values give the best collapse of the curves, as having been shown in Panels (a2), (b2), (c2) and (d2) of Figure 12.

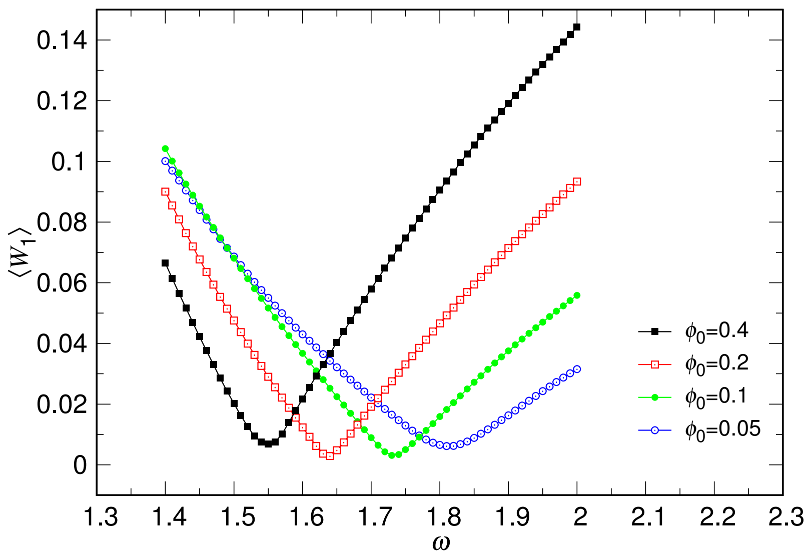

Figure. S4. Mean distribution width $\langle W_1 \rangle$ of the collapsed R' curves in the beginning t_ω region, calculated as a function of ω. The ϕ_0 values are indicated in the legend.
